# Ultra-Processed Food Intake Is Associated with Non-Alcoholic Fatty Liver Disease in Adults: A Systematic Review and Meta-Analysis

**DOI:** 10.3390/nu15102266

**Published:** 2023-05-10

**Authors:** Alex E. Henney, Conor S. Gillespie, Uazman Alam, Theresa J. Hydes, Daniel J. Cuthbertson

**Affiliations:** 1 Department of Cardiovascular & Metabolic Medicine, University of Liverpool, Liverpool L3 5TR, UK; ahenney@liverpool.ac.uk (A.E.H.); ualam@liverpool.ac.uk (U.A.);; 2 Metabolism & Nutrition Research Group, Liverpool University Hospitals NHS Foundation Trust, Liverpool L7 8XP, UK; 3 Department of Clinical Neurosciences, University of Cambridge, Cambridge CB2 1TN, UK; cg823@cam.ac.uk; 4 Department of Gastroenterology and Hepatology, Liverpool University Hospitals NHS Foundation Trust, Liverpool L7 8XP, UK

**Keywords:** ultra-processed food, NOVA, non-alcoholic fatty liver disease, non-alcoholic steatohepatitis

## Abstract

Non-alcoholic fatty liver disease (NAFLD) is associated with overweight/obesity, metabolic syndrome and type 2 diabetes (T2D) due to chronic caloric excess and physical inactivity. Previous meta-analyses have confirmed associations between ultra-processed food (UPF) intake and obesity and T2D. We aim to ascertain the contribution of UPF consumption to the risk of developing NAFLD. We performed a systematic review and meta-analysis (PROSPERO (CRD42022368763)). All records registered on Ovid Medline and Web of Science were searched from inception until December 2022. Studies that assessed UPF consumption in adults, determined according to the NOVA food classification system, and that reported NAFLD determined by surrogate (steatosis) scores, imaging or liver biopsy were included. The association between UPF consumption and NAFLD was assessed using random-effects meta-analysis methods. Study quality was assessed, and evidence credibility evaluated, using the Newcastle Ottawa Scale and NutriGrade systems, respectively. A total of 5454 records were screened, and 112 records underwent full text review. From these, 9 studies (3 cross-sectional, 3 case-control and 3 cohort), analysing 60,961 individuals, were included in the current review. Both moderate (vs. low) (pooled relative risk 1.03 (1.00–1.07) (*p* = 0.04) (I^2^ = 0%)) and high (vs. low) (1.42 (1.16–1.75) (<0.01) (I^2^ = 89%)) intake of UPF significantly increased the risk of NAFLD. Funnel plots demonstrate low risk of publication bias. Consumption of UPF is associated with NAFLD with a dose–response effect. Public health measures to reduce overconsumption of UPF are imperative to reduce the burden of NAFLD, and the related conditions, obesity and T2D.

## 1. Introduction

Non-alcoholic fatty liver disease (NAFLD) represents a disease spectrum ranging from liver fat accumulation (hepatic steatosis), an inflammatory hepatitis (non-alcoholic steatohepatitis, NASH) through to end-stage liver disease with fibrosis, cirrhosis and hepatocellular carcinoma [1]. Considered the hepatic component of metabolic syndrome (MetS), NAFLD is associated with related diseases such as type 2 diabetes (T2D) and obesity [2,3]. There is strong evidence that NAFLD is associated with an approximate twofold higher risk of developing T2D, irrespective of obesity and other common metabolic risk factors [4,5,6]. The accumulation of liver fat is associated with hepatic insulin resistance (with an impaired ability of insulin to suppress endogenous glucose production), hepatic inflammation and development of peripheral insulin resistance [7]. Furthermore, the critical effect of lifestyle modification, including a low-calorie diet and moderate physical activity [8] in improving liver health with significant improvements in hepatic steatosis, liver injury and fibrosis, is paralleled by prevention of progression to T2D (in people with impaired glucose regulation) [9] or remission of T2D, where diabetes has already developed. Newer glucose-lowering therapies for T2D also significantly benefit NAFLD [10]. Moreover, NAFLD prevalence mirrors that of T2D, with up to a third of adults affected [11], making it the most globally prevalent liver disease [12].

A significant pathophysiological driver of the burgeoning metabolic disease prevalence, and the increasing disparities and health inequalities seen among contrasting socioeconomic groups, is a dramatic transformation in the global food system with rapid growth of ultra-processed food (UPF) consumption [13]. UPFs are industrial formulations of cheap ingredients from high yield crops (such as refined sugar, starch, oil, protein isolates) and remnants of intense animal agriculture that are highly energy-dense due to total fat, saturated fat and trans-fat contributions, combined with low fibre and poor micronutrient profiles. They can be differentiated from processed foods due to additional chemical enhancement using preservatives, emulsifiers and artificial sweeteners aiming to increase shelf life and palatability [14]. They include such food items as confectionary sweets, high-sugar drinks and ‘microwave ready-meals’, constituting around half of daily energy intake in Western populations [15]. Their cheap production cost, contrasting with the higher relative cost of minimally processed foods, drives a high UPF consumption rate globally; particularly in low income households [16]. A further exacerbating factor is that their consumption promotes a cycle by further increasing caloric intake and progressive weight gain [17]. A myriad of food processing classification systems are developed to assess the processing level of food. However, a recent systematic review highlighted NOVA as the most specific, coherent, clear, comprehensive and workable of these systems [18].

To date, meta-analyses have demonstrated positive associations between UPF consumption and development of overweight/obesity [19] and related co-morbidities including T2D [20], cardiovascular disease (CVD) and cancer risk [21,22]. In addition, experimental study has demonstrated that UPFs promote development of inflammatory diseases via promotion of systemic inflammation [23]. While observational studies have demonstrated evidence to support a link between UPF and NAFLD [24,25,26,27], no meta-analysis has objectively explored the association.

The primary aim of our systematic review and meta-analysis is to assess and quantify the relationship between consumption of UPF and the prevalence of NAFLD. The secondary aim is to assess whether a dose–response relationship exists between UPF consumption and NAFLD.

## 2. Materials and Methods

The protocol for this review was registered on the International Prospective Register of Systematic Reviews (PROSPERO) (CRD42022368763).

### 2.1. Search Strategy and Selection Criteria

The methods constructed in the Preferred Reporting Items for Systematic Reviews and Meta-Analyses (PRISMA) guidelines were used to develop this review [28]. Medline and Web of Science were searched (AH and CG) on 5 December 2022 for all original research describing associations between UPF intake and development of NAFLD. The search algorithm used was comprised of two groups of keywords based on previous meta-analyses [19,20,25]. The first group included words related to UPF and the second group included words related to NAFLD. The Boolean operators ‘OR’ and ’AND’ were used to separate words within each individual group and between groups, respectively. The full search criteria are described in (Supplementary Table S1). We also performed manual searches of reference lists of relevant studies and review articles returned by the initial search, as well as contacted experts in the field, to identify any additional articles. No restriction was placed on the earliest search date and searches were performed up to the current date (December 2022).

### 2.2. Inclusion Criteria

To be included in the current systematic review and meta-analysis, the following criteria had to be met: (1) the study was observational (including prospective or retrospective cohort studies, case-control and cross sectional study designs); (2) exposure to UPF was assessed, either as the main exposure or as part of subgroup analysis. UPF intake needed to be assessed according to the NOVA food classification system, although we also included studies that did not directly reference the NOVA food classification system but evaluated foods according to its criteria; (3) assessed the association of UPF with NAFLD prevalence; (4) participants were adults (>18 years of age); (5) results were reported as either odds ratios (ORs), relative risk (RR), hazard ratios (HRs) or beta coefficient, or provided numbers for the calculation of such effect sizes; (6) NAFLD could be defined using steatosis scores (Fatty Liver Index (FLI) or Controlled Attenuation Parameter (CAP) scores from Fibroscan), imaging (ultrasonography (including transient elastography), CT or MRI/MRS) or liver biopsy. We did not include studies that defined NAFLD based on liver enzymes alone.

### 2.3. Exclusion Criteria

Studies were excluded from this current systematic review and meta-analysis if they were any of the following: (1) animal studies; (2) in vitro studies; (3) secondary research including other review articles; (4) studies that used non-adult (<18 years of age) populations; (5) studies that only focused on beverages, (6) case reports, editorials, abstracts, unpublished studies or practice guidelines.

### 2.4. Outcome

The primary outcome of the current study was the difference in prevalence of NAFLD between patients with low vs. high intake of UPF. Low intake of UPF, defined as the non-consumption or lowest consumption reported by each study, was considered the reference group. High intake of UPF was defined as the highest reported consumption value from each study. The secondary outcome was whether a dose–response association existed between UPF consumption and NAFLD, assessed by the difference in prevalence of NAFLD in patients with a low compared to those with moderate and high intake of UPF. We considered moderate intake of UPF to be the first exposure group in each study; most commonly the second quartile or tertile of intake. The exception to this rule was if intake was stratified by quintiles, in which case the third quintile, or second exposure group, was used.

### 2.5. Study Selection

Two reviewers (AH and CG) used the inclusion and exclusion criteria to select appropriate literature from Medline and Web of Science; using Rayyan to navigate the selection process. Articles were screened by titles and abstracts then subsequently full texts of selected articles were reviewed. Any disagreements were resolved via discussion between the two reviewers. In addition, the authors performed manual searches of reference lists of relevant studies and contacted experts in the field to highlight other articles not already identified. If an expert thought a currently unidentified paper was suitable for inclusion, this was discussed via video call between the first reviewer (AH) and the expert. The discussion involved whether or not the recommended paper met inclusion and exclusion criteria, as well as potential reasons for not being identified through systematic searches of databases.

### 2.6. Data Extraction

From the included studies, the following data were extracted for use in the current study by reviewer one (AH) and independently checked by reviewer two (CG): (1) basic study information (author name, year of publication, journal) and (2) study design, population, country, study type, sample size, follow up, adjustment for confounding variables, definitions used for UPF and NAFLD (including dietary assessment tool, whether the study evaluated UPFs by NOVA classification system directly or not, NAFLD diagnostic tool), study outcomes (reported risk estimates in relation to NAFLD development (OR, HR, RR or beta coefficient and 95% confidence intervals)). For studies that did not report the necessary data, corresponding authors were contacted for the relevant data.

### 2.7. Quality of Evidence

Newcastle Ottawa scale [29] was utilised to assess the quality of evidence. Newcastle Ottawa scale is a validated tool recommended by Cochrane to assess the quality of observational research. It is composed of eight items that evaluate study selection, comparability and outcome, with a maximum score of 9 stars available for the highest quality study. As per a recent meta-analysis comparing the association between UPF and T2D, we stratified evidence into three groups: low quality scored <5 stars, medium quality scored 5 or 6 stars and high-quality scored >6 stars [20].

In addition, the NutriGrade scoring system was used to assess the overall credibility of evidence. The tool is an eight-item scale that evaluates evidence for meta-analyses related to nutrition. The eight items are as follows: (1) risk of bias, study quality and limitations; (2) precision; (3) heterogeneity; (4) directness; (5) publication bias; (6) funding bias; (7) effect size and (8) dose–response. To interpret NutriGrade evaluation, the following scoring system was used: (a) very low (0–3.99); (b) low (4–5.99); (c) moderate (6–7.99); (d) high (8–10) [30].

### 2.8. Meta-Analysis

A random-effects model was used to calculate a pooled RR ± 95% CI from all the included studies because the extracted data was expected to be highly heterogeneous. The random effects model used was Cochran–Mantel–Haenzel test [31]. This was carried out using R Studio V4.0.2 (R Studio PBC, Boston, MA, USA, meta, ggplot2, and metafor packages). The Higgins I^2^ statistical technique was used to assess heterogeneity between the included studies [32], supported by the Paule–Mandel test to estimate tau^2^ [33]. To be considered highly heterogeneous, values were required to be >75% (*p* ≤ 0.05). We used the result from the fully adjusted model in the included studies when conducting all analyses. Summary-level meta-regression analysis was performed using a fixed-effects model to evaluate whether a dose–response association existed between increasing UPF intake and risk of NAFLD (metareg and bubble functions from meta package). A bubble plot was generated to represent these data.

Sensitivity analysis was performed using a random effects model to see whether results would change dependent on study design (longitudinal or non-longitudinal), reporting of UPF (NOVA or non-NOVA classified), sample size (<1000 or >1000 participants), continent (North America, Europe or Asia) or method of dietary assessment. Begg’s funnel plots were generated for visualisation of publication bias.

## 3. Results

### 3.1. Study Characteristics

**Figure 1:**
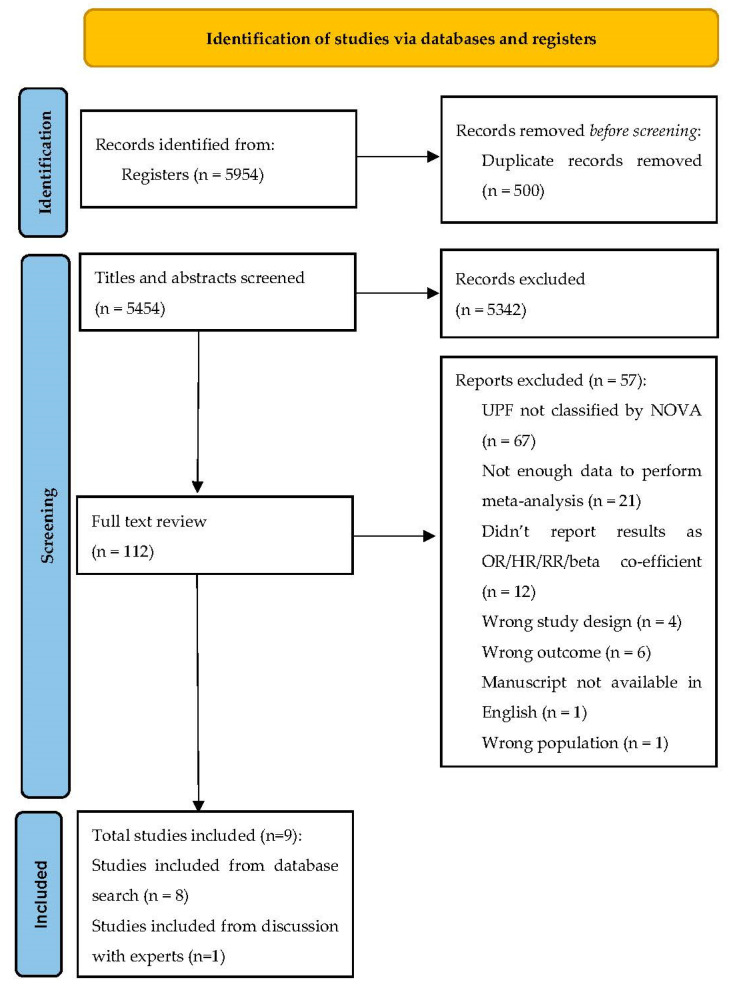
Preferred Reporting Items for Systematic Reviews and Meta-Analysis (PRISMA)-reported flow diagram for study selection process.

A flowchart demonstrating the selection process of the studies is illustrated in (Figure 1). After duplicates were excluded (*n* = 500), 5454 records were identified and 8 met inclusion criteria and were subsequently included in the current study. A further single article, not previously identified via systematic literature search, met the inclusion criteria following discussion with an expert in the field. In total, 60,961 participants were analysed. In full text review, most studies were excluded due to not classifying UPF via the NOVA classification system (whether NOVA was referenced directly or not) (*n* = 67) or lacking sufficient data to perform meta-analysis (*n* = 21).

The main demographics and results of the studies included in this systematic review and meta-analysis are shown in Table 1. The studies included participants from Asia [24], the USA [34,35] and Europe [25,26,27,36,37,38]. Three of the included studies were longitudinal, with follow up times ranging from 1 to 25 years [24,26,34], three were case-control [35,36,37], and three were cross-sectional in design [25,27,38]. Sample sizes ranged from 286 to 32,448 [35,36]. All nine studies included both males and females. Four studies directly evaluated UPFs using the NOVA classification system [24,25,26,27], with the other five studies assessing foods named as ultra-processed by the NOVA classification system. Eight of the studies collected dietary information via Food Frequency Questionnaires (FFQs) [24,25,26,27,35,36,37,38], whilst the last study used a semi-structured interview [34]. All of the studies were published within the last four years.

(Supplementary Table S2) shows the quality of evidence as reported by the NOS. The studies had NOS scores ranging from 7–9 with a mean of 8.1. All the studies included were considered high quality evidence. (Supplementary Table S3) shows the NutriGrade evaluation of credibility of evidence. The credibility of evidence in this systematic review and meta-analysis is considered high with an overall score of 8.

### 3.2. Systematic Review

Seven studies reported significant associations between UPF consumption and the development of NAFLD [24,26,34,35,36,37,38]. The two non-significant studies reported a trend towards increased risk [25,27]. Only three studies reported significant associations between UPF consumption and the development of NAFLD with moderate intake of UPFs [24,26,34] (Table 2).

### 3.3. Association between Ultra-Processed Food Intake and Non-Alcoholic Fatty Liver Disease

**Figure 2:**
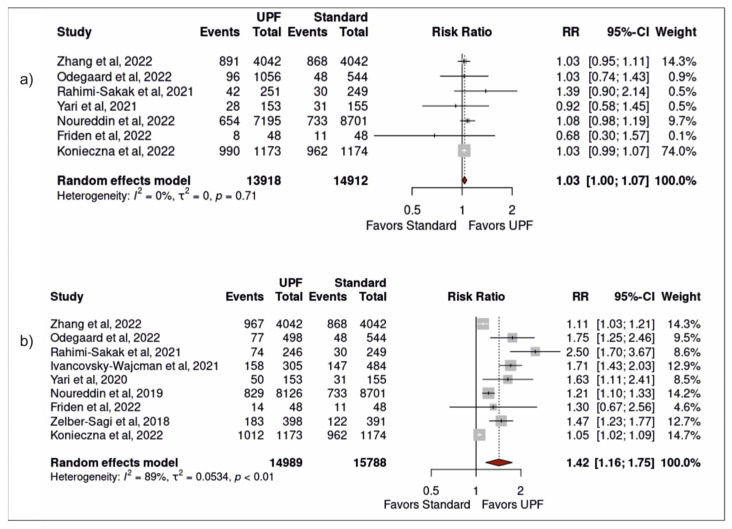
Forest plots from a random-effects model portraying the association between (**a**) moderate (vs. low) ultra-processed food intake and development of NAFLD; (**b**) high ultra-processed food intake and development of NAFLD [24,25,26,27,34,35,36,37,38].

**Figure 3:**
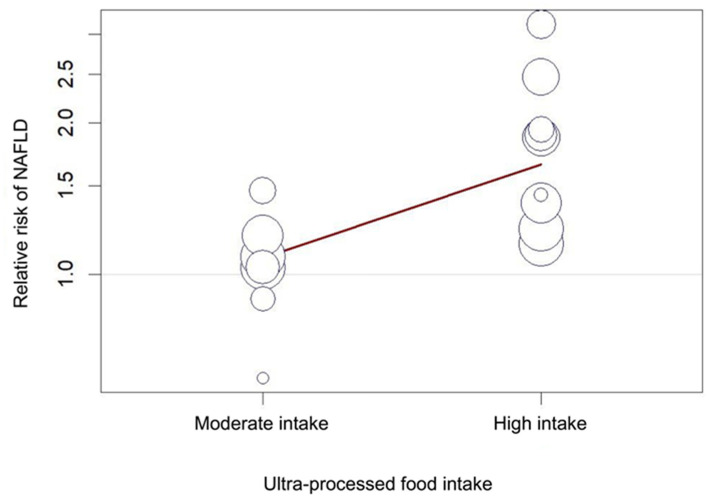
Bubble plot for meta-regression exploring the dose–response association between ultra-processed food intake and NAFLD. The size of circle represents the sample size of individual included the studies.

Moderate intake of UPFs was associated with increased risk of developing NAFLD (pooled RR 1.03 (1.00–1.07) (*p* = 0.04)), with low heterogeneity (I^2^ = 0% (*p* = 0.71)). Neither the study by Ivancovsky-Wajcman et al. nor the study by Zelber-Sagi et al. included data for moderate intake of UPFs and they were therefore excluded from this meta-analysis [25,38] (Figure 2a). High intake of UPFs was associated with increased risk of developing NAFLD (1.42 (1.16–1.75) (<0.01)), with significant heterogeneity (89% (<0.01)) (Figure 2b). A bubble plot was used to visually represent the dose–response effect between UPF intake and NAFLD following meta-regression analysis (Figure 3).

### 3.4. Sensitivity Analysis

Sensitivity analyses have been summarised in (Supplementary Table S4).

**Figure 4:**
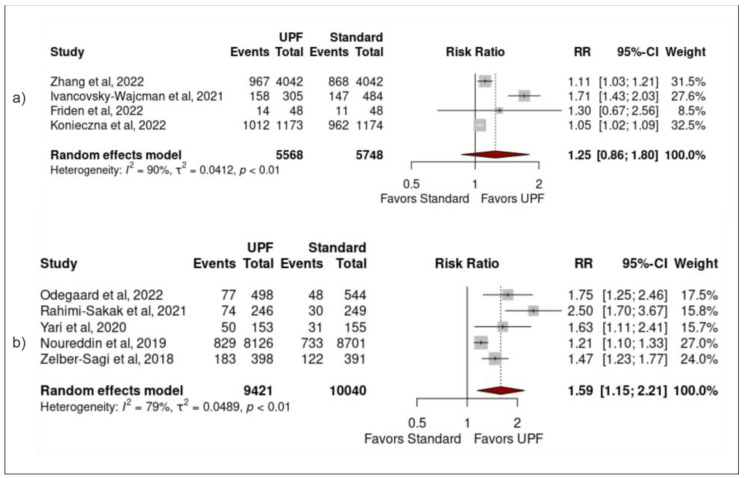
Forest plots demonstrating the association between high UPF intake and NAFLD when studies (**a**) directly referenced NOVA, (**b**) did not directly reference NOVA [24,25,26,27,34,35,36,37,38].

UPF classification: Significant associations remained between high UPF intake and NAFLD when sensitivity analysis was performed using only studies that did not directly reference use of NOVA (1.59 (1.15–2.21) (0.02)), but no significant result was found using only studies that did reference NOVA (1.25 (0.86–1.80) (0.17)) (Figure 4).

**Figure 5:**
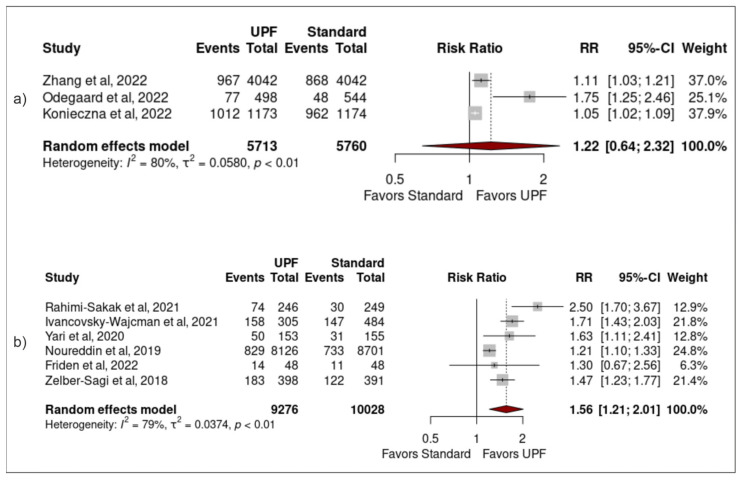
Forest plots demonstrating the association between high UPF intake and NAFLD when studies (**a**) were longitudinally designed, (**b**) were not longitudinally designed [24,25,26,27,34,35,36,37,38].

Study design: Significant associations remained between high UPF intake and NAFLD when sensitivity analysis was performed using only studies that were non-longitudinally designed (1.56 (1.21–2.01) (<0.01)), but no significant result was found using only studies that were longitudinally designed (1.22 (0.64–2.23) (0.41)) (Figure 5).

**Figure 6:**
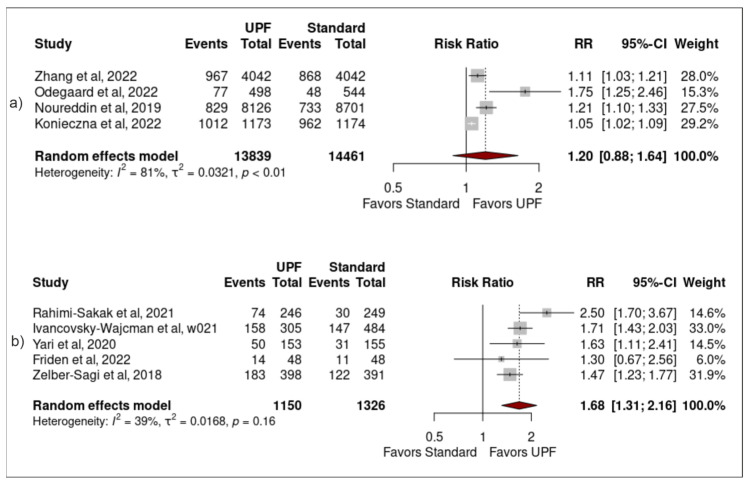
Forest plots demonstrating the association between high UPF intake and NAFLD when studies (**a**) had sample size greater than 1000 participants, (**b**) had sample size less than 1000 participants [24,25,26,27,34,35,36,37,38].

Sample size: Significant associations remained between high UPF intake and NAFLD when sensitivity analysis was performed using only studies that had sample sizes of less than 1000 participants (1.68 (1.31–2.16) (<0.01)), but no significant result was found using only studies that had sample sizes greater than 1000 participants (1.20 (0.88–1.64) (0.16)) (Figure 6).

In addition, when sensitivity analyses were performed based on geographical location of study, studies that were based in Europe (1.52 (1.12–2.07) (0.02)) or the rest of the world (1.44 (1.10–1.89) (0.02)) both resulted in significant results. Moreover, studies that used food frequency questionnaires (FFQs) 1.40 (1.11–1.76) (0.01) demonstrated significant associations between intake of UPFs and NAFLD. Corresponding forest plots are found in (Supplementary Figures S1–S4).

### 3.5. Publication Bias

**Figure 7:**
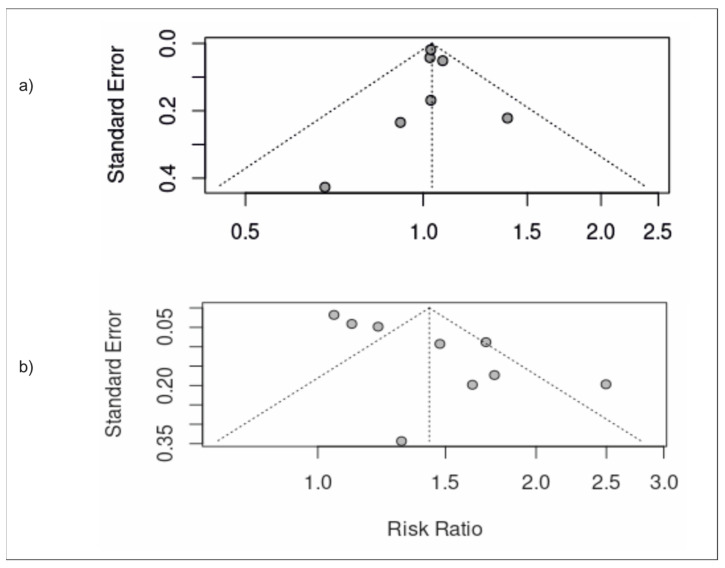
Funnel plots portraying risk of publication bias for studies assessing the association between (**a**) moderate ultra-processed food intake and development of NAFLD; (**b**) high ultra-processed food intake and development of NAFLD.

Begg’s funnel plots were used to visually represent publication bias among the included studies. Funnel plots were largely symmetrical suggesting low risk of publication bias (Figure 7).

## 4. Discussion

We present the first systematic review and meta-analysis assessing the association between UPF consumption and NAFLD prevalence and demonstrate a striking association between the consumption of UPFs and the development of NAFLD, with a clear dose–response relationship. The risk of NAFLD increases in proportion to the quantity of UPF consumption.

The association of obesity, NAFLD and T2D with UPFs is unsurprising; UPFs are characterised by a poor nutritional profile combining high energy density, total fat, saturated fat, refined carbohydrate and salt composition with a low composite of fibre, vitamins and minerals [39]. This association with NAFLD is likely multi-factorial [19] relating to a myriad of mechanisms including increased total daily energy intake, adverse macronutrient and micronutrient composition, additional harmful additive chemicals during the processing stage and the presence of other lifestyle-related factors such as physical inactivity.

**Figure 8:**
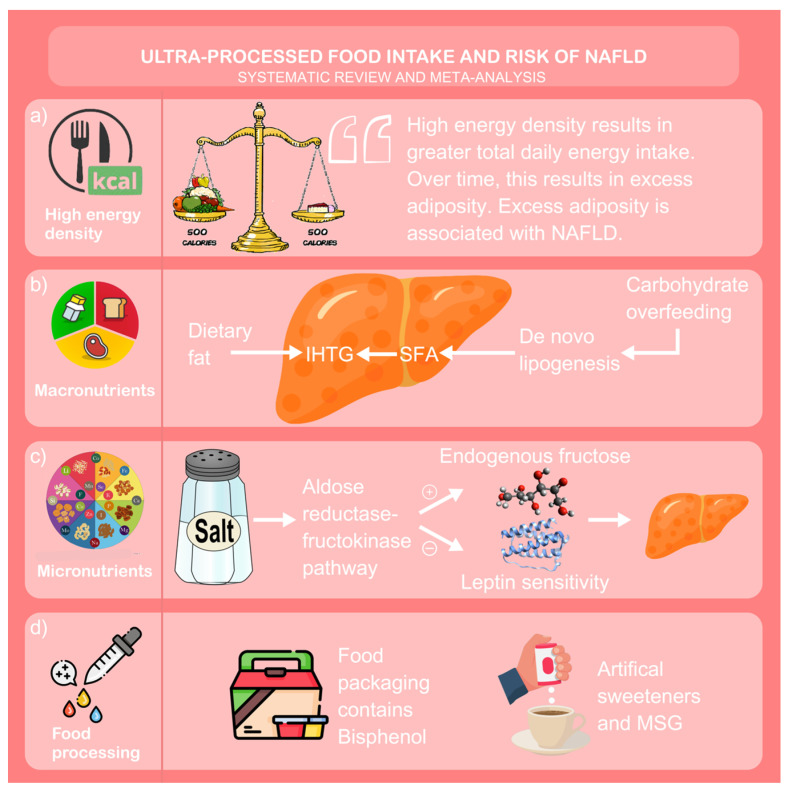
Graphical discussion. NAFLD = non-alcoholic fatty liver disease; IHTG = intra-hepatic triglyceride deposition; SFA = saturated fatty acids; MSG = monosodium glutamate. (**a**) ultra-processed foods are highly energy dense, contributing towards excess visceral adiposity and fatty liver disease; (**b**) ultra-processed foods are high in dietary saturated fat and refined carbohydrates which promote de novo lipogenesis and consequent intra-hepatic triglyceride deposition if consumed in excess, chronically; (**c**) ultra-processed foods are high in sodium salt which activates the aldose reductase-fructokinase pathway. This in turns upregulates endogenous fructose availability and downregulates leptin sensitivity, increasing hepatic adiposity; (**d**) ultra-processed foods are often saturated with artificial food processing ingredients in their food matrix, such as artificial sweeteners and mono-sodium glutamate, and packaging, often in the form of bisphenol a.

Obesity: The association between obesity and NAFLD has been well-described historically, with increasing BMI independently predicting NAFLD risk [40]. UPFs can contribute towards excess visceral adiposity in a number of ways. Firstly, UPFs are energy dense; resulting in a higher daily energy intake which contributes towards excess adiposity [41]. A recent systematic review and meta-analysis assessing the association between diet and NAFLD found that total energy intake was the only dietary factor driving development of NAFLD [42]. However, Tsompanaki et al. did not address the issue of ultra-processing in their review [42]. In fact, except for the studies by Friden et al. and Konieczna et al. [26,27], all of the included studies in our review adjusted for total energy intake in their final analytical model. In addition, UPFs appear to be associated with obesity independent of total energy intake [19]. Thus, we suggest that UPFs likely contribute to obesity through a variety of additional mechanisms. For example, UPF consumption is associated with rapid gastric emptying by virtue of lower fibre content and consequent altered gut hormone signalling to satiety pathways in the central nervous system [43]. In addition, the high refined carbohydrate and saturated fat composition of UPFs can lead to derangement in the neurocircuit involved in appetite regulation, encouraging overfeeding [44] (Figure 8). Despite the above, excluding the studies again by Friden et al. and Konieczna et al. [26,27], all of the included studies in our review adjusted for BMI and hence other mechanistic explanations need to be explored to address the association between UPFs and NAFLD.

Macronutrient composition: The macronutrient profile of UPFs is characterised by high total fat, saturated fat and free sugars from refined carbohydrates, as well as low fibre [45]. High dietary fat intake promotes intra-hepatic triglyceride (IHTG) deposition, although this is influenced by the type of dietary fat ingested: saturated fats promote, and polyunsaturated fats reduce hepatic fat deposition. Additionally, high fat inhibits antioxidant activity. Combined with high fat is excessive intake of carbohydrates, such as fructose, which promote hepatic de novo lipogenesis. Chronic, recurrent episodes of lipogenesis increase IHTG deposition [46,47,48]. Low-quality carbohydrates, such as those found in UPFs, are typically lower in fibre. High fibre intake is protective against liver fat deposition, not only by reducing total energy intake, but also by encouraging a healthy gut microbiota that deters chronic inflammation and liver injury [49]. The potential damaging effect of macronutrients on liver fat is highlighted well by the NOVA group 4 product, sugar sweetened beverages (SSBs). Despite having low energy density, SSBs are associated with a higher risk of NAFLD, T2D and obesity [50,51]; partly explained by addition of low-quality carbohydrate such as fructose [46]. Fructose drives development of NAFLD through increased transcription of carbohydrate-responsive element-binding protein and increased uric acid generation following metabolism by fructokinase C [52,53]. In addition, SSBs stimulate appetite through secretion of orexigenic hormones, resulting in further carbohydrate ingestion [54]. Interestingly, Tsompanaki et al. did not find an association between SSBs and NAFLD in their review, however this is likely explained by their classification of intake being ‘no intake’ or ‘any intake’ which may dilute the effects of higher SSB consumption [42] (Figure 8).

Micronutrient composition: UPFs contain unhealthy levels of micronutrients such as sodium salt [45]. A recent meta-analysis demonstrated an association between high salt intake and NAFLD [55]. It was suggested salt contributed towards NAFLD development through the salt-induced aldose reductase–fructokinase pathway in the hypothalamus and liver, as well as dysregulation of the renin-angiotensin system (RAS). Activation of the aldose reductase–fructokinase pathway increases endogenous fructose turnover and enhances leptin resistance, both of which are associated with visceral adiposity [55] (Figure 8).

Although it is becoming more apparent that UPFs exert damaging effects on the liver through more than just their promotion of obesity, between-study heterogeneity in our review was high, in part due to a lack of standardisation in defining UPFs. Only four studies directly referenced NOVA when evaluating the association between UPFs and NAFLD, whereas five reported UPF intake indirectly of NOVA. This meant that the focus varied between specific types of UPFs, such as processed meats, fast food or energy-dense snacks, and an overall dietary pattern. Surprisingly, during sensitivity analysis, there was no significant association between UPF consumption and NAFLD when using only the four studies reporting UPF intake via NOVA classification directly. This is most likely owing to a lack of power although it could also suggest that ultra-processing of specific foods may increase NAFLD risk more than others, highlighting a need for more primary research using NOVA as a standardised method to define UPFs. For example, processed meats are rich in nitrites that induce oxidative stress, lipid peroxidation and pro-inflammatory cytokine release [35,37,38]. Nitrites can also form nitrosamines when cooked at high temperatures which are associated with diseases of insulin resistance [56]. Furthermore, various studies have highlighted that diets higher in animal protein (compared to other protein from other plants) are at higher risk of NAFLD [38]. Despite total protein intake not varying between patients with NAFLD and controls, the review by Tsompanaki et al. was not able to highlight whether differences in protein origin bear weight on development of NAFLD and this could be an area for future research [42]. Whether the types of UPFs consumed dictate the degree of fatty liver promotion is particularly important when considering strategies to reduce UPF consumption on a population level, as participants in different countries may consume different kinds of UPF, although our study suggests that associations largely remain significant independent of participant geography.

Non-nutritional factors: Despite experimental research highlighting probable associations between adverse macro- and micronutrient profiles with NAFLD, the review by Tsompanaki et al. concluded that neither of these dietary components were associated NAFLD [42] and hence other explanations should also be sought. However, that review was limited by not examining non-nutritional components of food. The manufacturing process behind UPFs is metabolically damaging through chemical modification, cosmetic additives and artificial packaging [14]. Common cosmetic additives to UPF, such as monosodium glutamate and artificial sweeteners, have been associated with NAFLD [57,58] and other components of the MetS [59], mediated by alteration to the gut microbiome to a pro-inflammatory environment [60]. Bisphenol, a common ingredient in artificial UPF packaging, is associated with greater incidence and progression of NAFLD due to endocrine disruption and the pleiotropic action of the compound [61] as well as increasing the risk of other metabolic diseases such as T2D [62]. Finally, timing of eating impacts metabolic outcomes, with high consumption of low-quality carbohydrates, such as those found in UPFs, being more harmful when consumed during evening meals [63] (Figure 8).

Strategies employed so far to reduce UPF intake include taxation to UPFs and using this taxation to subsidise the cost of more minimally processed foods [64,65]. A handful of countries are leading the way with fiscal policies that aim to tackle this issue [66]. Mexico leads the way on sugar-based taxes on sweetened beverages and packaged foods which demonstrated taxation significantly reduced consumption of these foods in line with the level of tax [67,68]. Other countries such as the United Kingdom and South Africa quickly followed suit.

### Strengths and Limitations

This systematic review and meta-analysis is the first to objectively evaluate the association between UPF consumption and NAFLD prevalence, adding to the literature from the nine included studies. Secondly, we performed an extensive literature search including searching two large databases, Ovid Medline and Web of Science, while performing a non-systematic search of reference lists and contacting experts in the field to find all available relevant data. Thirdly, our search terms enabled us to identify any study that has evaluated UPFs according to NOVA classification.

A limitation of all meta-analyses is reliance on the quality of included studies reflecting sometimes a paucity of evidence. With only nine studies meeting inclusion criteria, the representativeness of our findings may not be translatable to all populations. Between-study heterogeneity was high due to discrepancy in study design, method of dietary assessment, method of NAFLD diagnosis and quantification of UPFs. For example, only three eligible studies assessed the association between UPF and NAFLD longitudinally and the association between UPFs and NAFLD lost significance when meta-analysing only these studies. This is most likely explained by insufficient power, but cross-sectional studies may exaggerate the association due to inherently higher bias. Most studies evaluated diet using FFQs which risk recall bias and underreporting of true intake [69]. Cross-sectional studies included in the current review may not have been an accurate record of long-term habitual dietary intake. Five of the studies used ultrasonography or Fibroscan to diagnose NAFLD. Ultrasonography is a widely available imaging tool used to screen asymptomatic patients who have deranged liver enzymes for features of fatty liver. However, the sensitivity of ultrasonography for NAFLD is limited, being unable to distinguish between different types of NAFLD or stage hepatic fibrosis [70]. It would also be beneficial to assess whether the severity of NAFLD was associated with increasing UPF intake using these more sensitive imaging modalities. Moreover, each study used its own definition for quantity of UPF intake, rather than a pre-determined cut-off point, and this resulted in a variety of different exposure groups being reported from low vs. high binary grouping through to tertiles, quartiles and quintiles of intake using either absolute intake, percentage of total daily energy intake or weight in grams. This meant it was hard to determine whether a high intake of UPFs in one country is the equivalent to moderate intake in another. We would suggest more longitudinal research is needed in this area to draw more firm conclusions here using more sensitive imaging and a standardised approach to define UPFs in research using NOVA.

## 5. Conclusions

The current systematic review and meta-analysis produced consistent findings for the association between UPFs and NAFLD with a dose–response effect and provides valuable evidence to support public health policies in respect to dietary advice in this increasingly prevalent disease. We would advocate global efforts to minimise consumption of UPFs, in exchange for fresh and minimally processed foods, with promotion of physical activity to tackle the societal burden of this disease.

## Figures and Tables

**Table 1:** Description of studies included in the systematic review (*n* = 9).

ID	Sample	Population (SEX/AGE)	Study Design (Follow up)	Exposure (Via NOVA Unless Stated Otherwise) (Moderate vs. High Intake of Upf)	Adjustment	Outcome (Risk of Nafld)
Zhang et al. [24]	Tianjin Chronic Low-grade Systemic Inflammation and Health (TCLSIH) Cohort Study	16,168 males/females aged 18–90 years	Cohort (4.2 years)	Moderate: 2° quartile (30.1g/1000 kcal per day) High: 4° quartile (113.7 g/1000 kcal per day)	Age, sex, BMI, smoking, alcohol, education, occupation, monthly household income, physical activity, family history of cardiometabolic disease, depressive symptoms, total energy intake, healthy diet score, diabetes, hypertension and hyperlipidaemia	Moderate intake increased the risk by 13% High intake increased the risk by 18%
Odegaard et al. [34]	Coronary Artery Risk Development in Young Adults (CARDIA) study	3001 male/females, aged 24–29 years	Cohort (25 years)	Fast-food: Moderate: 3° quintile (1–2x/week) High: 5° quintile (>3x/week)	Age, sex, race, study centre, education, employment history, household income, smoking, alcohol, diet quality, energy intake, physical activity, and prevalence of type 2 diabetes or history of a CVD event at the year 25 exam	Moderate intake increased the risk by over two-fold High intake increased the risk by over five-fold
Yari et al. [36]	Iranian males and females	614 male and females, mean age 38.92 years	Case-control study	Energy-dense nutrient- poor snacks: Moderate: 2° quartile (3.7% total energy intake) High: = 4° quartile (9.7% total energy intake)	Age, sex, BMI, physical activity, alcohol and total energy intake	Moderate intake had no significant association High intake increased the risk by over two-fold
Rahimi-Sakak et al. [37]	Iranian males and females	999 males/females, mean age of 43.54 years	Case-control study	Processed meat: Moderate: 2° quartile (0.4–2.4/day) High: 4° quartile (>6.6 g/day)	Age, gender, BMI, total energy intake, dietary factors, diabetes, smoking, and physical activity	Moderate intake had no significant association High intake increased risk by over three-fold
Noureddin et al. [35]	The Multi-ethnic Cohort (MEC) study	32,448 males/females, mean age 57.7 years	Nested case-control study	Processed meat: Moderate: 2° quartile (1.6–3.3 g/day) High: 4° quartile (>5.7 g/day)	BMI, alcohol intake, coffee drinking, total sweetened beverage intake, physical activity, total energy intake, education, smoking status and cardiovascular disease	Moderate intake had no significant association High intake increased the risk by 18%
Ivancovsky-Wajcman et al. [25]	Israeli males and females	789 males/females, mean age 58.83 years	Cross-sectional study	Moderate: no data High: >28% total energy intake	Age, gender, BMI, SFA intake, protein intake as a percentage of total energy intake, physical activity, coffee drinking and fibre intake	High intake had no significant association
Friden et al. [27]	Prospective investigation of Obesity, Energy and Metabolism (POEM)	286 males/females, age-matched at 50 years	Cross-sectional study	Moderate: 2° tertile (37.6% total energy intake) High: 3° tertile (49% total energy intake)	Sex, education level, physical activity level, smoking status, dietary factors and BMI	Moderate or high intake had no significant association
Zelber-Sagi et al. [38]	Colonoscopy screening at the Department for Gastroenterology and Hepatology at Tel Aviv Medical Centre	789 males/females, mean age 58.83 years	Cross-sectional study	Processed meat: Moderate: no data available High: >0.33 daily portions	Age, gender, energy intake per day, BMI, weekly hours of physical activity, smoking status, weekly alcohol portions, saturated fat (percent of daily energy) and cholesterol intake	High intake increased the risk by 47%
Konieczna et al. [26]	PREDIMED-Plus trial	5867 males/females, mean age 65.0 years	Cohort (1 year)	Moderate: 3° quintile (6.23% of g/day) High: 5° quintile (19% of g/day)	Age at inclusion, sex, study arm, and follow-up time (months), baseline educational level, smoking habits, alcohol intake	Moderate intake was associated with a two-fold increased likelihood High intake was associated with four-fold increased likelihood

**Table 2:** Summary of results of included studies. All results are taken from fully adjusted models. FFQ = food frequency questionnaire; US = ultrasound; FLI = Fatty Liver Index; HU = Hounslow Unit; CAP = Controlled Attenuation Parameter.

ID	Dietary Assessment Tool	Nafld Diagnostic Tool (Nafld Criteria)	Odds Ratio (or) or Beta Coefficient (BC)	Effect Size (Relative RISK (RR))
Zhang et al. [24]	FFQ	US abdomen: (any two of: (a) increased echogenicity; (b) deep attenuation of signal; (c) vascular blurring)	Moderate: (OR 1.13 (1.03–1.25) (*p* = <0.01)) High: (OR 1.18 (1.07–1.30) (*p* = <0.01))	Moderate: (RR 1.03 (0.95–1.11)) High: (RR 1.11 (1.03–1.21))
Odegaard et al. [34]	Semi-structured interview	CT abdomen: (liver attenuation < 40 HU)	Moderate: (OR 2.31 (1.34–3.98) (*p* = <0.01)) High: (OR 5.18 (2.87–9.37) (*p* = <0.01))	Moderate: (RR 1.03 (0.74–1.43)) High: (RR 1.75 (1.25–2.46))
Yari et al. [36]	FFQ	Fibroscan: (CAP score > 263)	Moderate: (OR 0.92 (0.48–1.77)) High: (OR 2.27 (1.19-4.31) (*p* = <0.01))	Moderate: (RR 0.92 (0.58–1.45)) High: (RR 1.63 (1.11–2.41))
Rahimi-Sakak et al. [37]	FFQ	Fibroscan: (CAP score > 263)	Moderate: (OR 1.72 (0.84–3.52)) High: (OR 3.42 (2.16-5.43) (*p* = <0.01))	Moderate: (RR 1.39 (0.90–2.14)) High: (RR 2.50 (1.70–3.67))
Noureddin et al. [35]	FFQ	US abdomen: (standardised criteria)	Moderate: (OR 1.03 (0.02–1.16)) High: (OR 1.18 (1.05–1.32) (*p* = <0.01))	Moderate: (RR 1.08 (0.98–1.19)) High: (RR 1.21 (1.10–1.33))
Ivancovsky-Wajcman et al. [25]	FFQ	US abdomen: (standardised criteria)	High: (OR 1.12 (0.78–1.59) (*p* = 0.55))	High: (RR 1.71 (1.43–2.03))
Friden et al. [27]	FFQ	MRI: (hepatic fat content > 5.5%)	High: (OR 1.32 (0.84–2.09) (*p* = 0.23))	Moderate: (RR 0.68 (0.30–1.57)) High: (RR 1.30 (0.67–2.56))
Zelber-Sagi et al. [38]	FFQ	US abdomen: (standardised criteria)	High: (OR 1.47 (1.04–2.09) (*p* = 0.031))	High: (RR 1.47 (1.23–1.77))
Konieczna et al. [26]	FFQ	FLI: (score > 60)	Moderate: (BC 2.01 (1.46–2.55) (*p* = <0.001)) High: (BC 3.73 (3.10-4.35) (*p* = <0.001))	Moderate: (RR 1.03 (0.99–1.07)) High: (RR 1.05 (1.02–1.09))

## Data Availability

All data available in individual included studies.

## Supplementary Materials

The following supporting information can be downloaded at: https://www.mdpi.com/article/10.3390/nu15102266/s1.
