# Supplementary material for: Ultra-Processed Food Intake Is Associated with Non-Alcoholic Fatty Liver Disease in Adults: A Systematic Review and Meta-Analysis
Source: Nutrients. 2023 May 10;15(10):2266. doi: 10.3390/nu15102266 (PMC10224355; doi:10.3390/nu15102266)
Supplement: Supplementary file 1 [file nutrients-15-02266-s001.zip › nutrients-2358177-supplementary.pdf]

Supplementary Materials

ITEM	SCORE
**UPF**	Ultra-processed or ultraprocessed or fast foods or processed food or ultraprocessed food or ultra-processed food or processed meat or ultra processed food or ham or sausages or hamburger or bacon or luncheon meats or ready-to-eat or ready-to-consume or industrialized or fast-food or fast food or fastfood or junk food or prepared food or candy or ice cream or chocolate or snacks or hot dog or burger or dietary patterns or dietary behaviors or dietary habits or ultra processed or NOVA or NOVA system or NOVA classification
**NAFLD**	NAFLD or non-alcoholic fatty liver disease or non alcoholic fatty liver disease or fatty liver disease or fatty liver or NASH or non-alcoholic steatohepatitis

Supplementary Table S1: *Table of search terms*

a)

ID	REPRESENTATIVENESS OF EXPOSED COHORT	SELECTION OF NON-EXPOSED COHORT	ASCERTAINMENT OF EXPOSURE	OUTCOME OF INTEREST NOT PRESENT AT START OF STUDY	COMPARABILITY OF COHORTS	ASSESSMENT OF OUTCOME	FOLLOW UP LENGTH	FOLLOW UP RATE	SCORE
**(Zhang et al., 2022)**	A	A	B	A	A	A	A	B	9
**(Odegaard et al., 2022)**	B	A	B	B	A	A	A	B	8
**(Konieczna et al., 2022b)**	A	A	B	B	A	B	B	D	7

b)

ID	ADEQUACY OF CASE DEFINITION	REPRESENTATIVENESS OF CASES	SELECTION OF CONTROLS	DEFINITION OF CONTROLS	COMPARABILITY OF CASES AND CONTROLS	ASCERTAINMENT OF EXPOSURE	SAME METHOD FOR CASES AND CONTROLS	RESPONSE RATE	SCORE
**(Yari et al., 2020)**	A	A	B	A	A	B	A	A	9
**(Rahimi-Sakak et al., 2022)**	A	A	B	A	A	B	A	A	9
**(Noureddin et al., 2020)**	A	A	A	B	A	B	A	A	8

c)

ID	REPRESENTATIVENESS OF SAMPL	SAMPLE SIZE	RESPONSE RATE	ASCERTAINMENT OF EXPOSURE	COMPARIBILITY OF SUBJECTS IN DIFFERENT OUTCOME GROUPS	ASCERTAINMENT OF OUTCOME	STATISTICS	SCORE
**(Ivancovsky-Wajcman et al., 2021)**	B	A	A	B	A	B	A	8
**(Fridén et al., 2022)**	B	B	A	A	A	B	A	8
**(Zelber-Sagi et al., 2018)**	B	B	C	B	A	B	A	7

Supplementary table S2: **a)** *Quality of evidence of longitudinally designed studies as reported by Newcastle Ottawa Scale (NOS) (n=2),* **b)** *quality of evidence of case-control studies as reported by Newcastle Ottawa Scale (NOS) (n=3),* **c)** *quality of evidence of cross-sectionally designed studies as reported by an adapted version of Newcastle Ottawa Scale (NOS) (n=1).*

ITEM	SCORE
**1**	2
**2**	1
**3**	0.5
**4**	1
**5**	0.5
**6**	1
**7**	1
**8**	1
**TOTAL SCORE**	8
**CREDIBILITY**	High

Supplementary table S3: *Credibility of evidence as reported by the NutriGrade tool (n=7). Item 1 risk of bias, study quality and limitations (0-2 points) - Quality of evidence as per Newcastle Ottawa Scale (mean) 7 or above = 2 points; Item 2 precision (0-1 point) - ≥500 events and the 95% CI excludes the null value; or ≥500 events, but 95% CI overlaps the null value, and 95% CI excludes important harm (RR <1.2) = 1 point; Item 3 heterogeneity (0-1 point) - 2 to 5 studies = 0 points, ≥10 studies, heterogeneity measures adequately reported, random-effects models, and subgroups analyses were conducted = 1 point; Item 4 directness (0-1 point) - no important differences in the population or intervention; hard clinical outcome = 1 point; Item 5 publication bias (0 to 1 point) - <5 studies = 0 points; no evidence for publication bias with test or plot (≥10 studies) = 1 point; Item 6 funding bias (0 to 1 point) - report from academic or research institution = 1 point; Item 7 effect size (0 to 2 points) - RR >1.20 and corresponding test statistically significant (highest vs lowest category) = 1 point; Item 8 dose-response (0 to 1 point) - no dose-response analysis = 0 point; significant linear dose-response relation = 1 point.*

SENSITIVITY ANALYSIS	POOLED EFFECT SIZE (RR (95% CI) (P))	HETEROGENEITY (I^2 = 95% (P)) (%)
REPORTED AS NOVA DIRECTLY	1.25 (0.86-1.80) (0.17)	90 (<0.01)
NOT REPORTED AS NOVA DIRECTLY	1.59 (1.15-2.21) (0.02)	79 (<0.01)
LONGITUDINALLY DESIGNED	1.22 (0.64-2.32) (0.41)	80 (0.01)
NON-LONGITUDINALLY DESIGNED	1.56 (1.21-2.01) (<0.01)	79 (<0.01)
SAMPLE SIZE >1000	1.20 (0.88-1.64) (0.16)	81.2 (<0.01)
SAMPLE SIZE <1000	1.68 (1.31-2.16) (<0.01)	39 (0.16)
NORTH AMERICA	1.40 (0.14-13.98) (0.31)	76 (0.04)
EUROPE	1.52 (1.12-2.07) (0.02)	92 (<0.01)
REST OF THE WORLD	1.44 (1.10-1.89) (0.02)	90 (<0.01)
DIETARY ASSESSMENT TOOL	1.40 (1.11-1.76) (0.01)	89 (<0.01)

Supplementary Table S4*: Table summarising the findings of sensitivity analysis.*

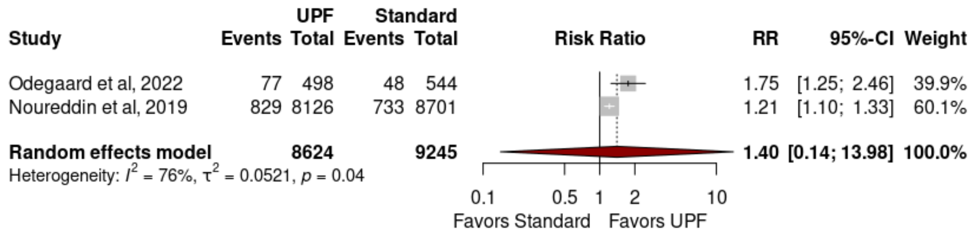

Supplementary Figure S1: *Forest plot portraying assessing the association between high ultra-processed food intake and development of NAFLD in studies conducted in North America only*

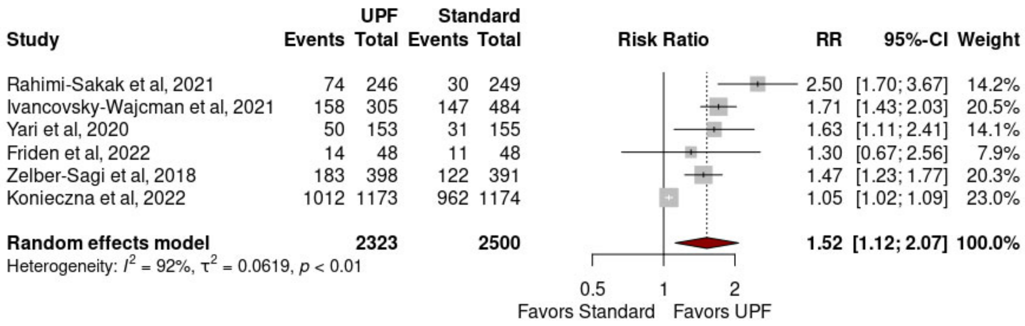

Supplementary Figure S2: *Forest plot portraying assessing the association between high ultra-processed food intake and development of NAFLD in studies conducted in Europe only*

Supplementary Figure S3: Forest plot assessing the association between high ultra-processed food intake and development of NAFLD in studies conducted in Europe and Asia, defined as 'rest of the world'

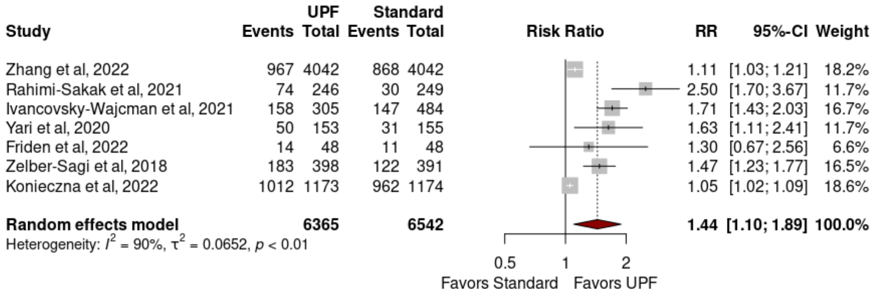

Supplementary Figure S4: Forest plot assessing the association between high ultra-processed food intake and development of NAFLD in studies using food frequency questionnaires as the dietary assessment tool of choice

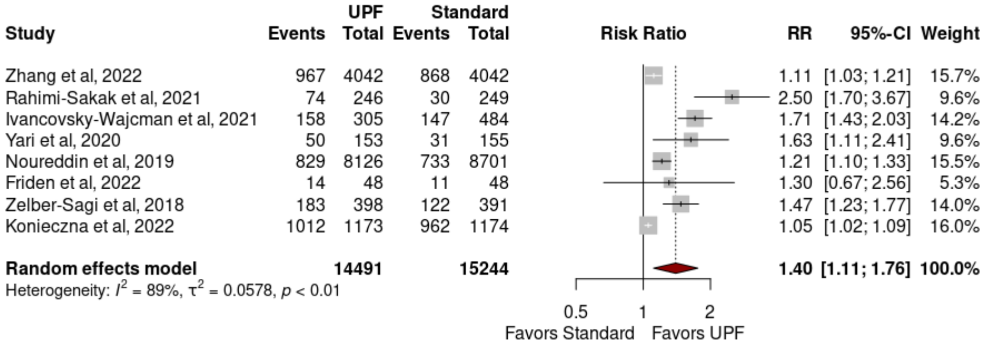

Section and Topic	Item #	Checklist item	Reported (Yes/No)
**TITLE**			
Title	1	Identify the report as a systematic review.	Yes
**BACKGROUND**			
Objectives	2	Provide an explicit statement of the main objective(s) or question(s) the review addresses.	Yes
**METHODS**			
Eligibility criteria	3	Specify the inclusion and exclusion criteria for the review.	Yes
Information sources	4	Specify the information sources (e.g. databases, registers) used to identify studies and the date when each was last searched.	Yes
Risk of bias	5	Specify the methods used to assess risk of bias in the included studies.	Yes
Synthesis of results	6	Specify the methods used to present and synthesise results.	Yes
**RESULTS**			
Included studies	7	Give the total number of included studies and participants and summarise relevant characteristics of studies.	Yes
Synthesis of results	8	Present results for main outcomes, preferably indicating the number of included studies and participants for each. If meta-analysis was done, report the summary estimate and confidence/credible interval. If comparing groups, indicate the direction of the effect (i.e. which group is favoured).	Yes
**DISCUSSION**			
Limitations of evidence	9	Provide a brief summary of the limitations of the evidence included in the review (e.g. study risk of bias, inconsistency and imprecision).	Yes
Interpretation	10	Provide a general interpretation of the results and important implications.	Yes
**OTHER**			
Funding	11	Specify the primary source of funding for the review.	NA
Registration	12	Provide the register name and registration number.	NA

Supplementary table S5: PRISMA abstract checklist

Section and Topic	Item #	Checklist item	Location where item is reported
**TITLE**			
Title	1	Identify the report as a systematic review.	Title page
**ABSTRACT**			
Abstract	2	See the PRISMA 2020 for Abstracts checklist.	Page 3
**INTRODUCTION**			
Rationale	3	Describe the rationale for the review in the context of existing knowledge.	Page 6
Objectives	4	Provide an explicit statement of the objective(s) or question(s) the review addresses.	Page 6
**METHODS**			
Eligibility criteria	5	Specify the inclusion and exclusion criteria for the review and how studies were grouped for the syntheses.	Page 7-8
Information sources	6	Specify all databases, registers, websites, organisations, reference lists and other sources searched or consulted to identify studies. Specify the date when each source was last searched or consulted.	Page 6-7
Search strategy	7	Present the full search strategies for all databases, registers and websites, including any filters and limits used.	Page 6-7 and supplementary material (supplementary table S1)
Selection process	8	Specify the methods used to decide whether a study met the inclusion criteria of the review, including how many reviewers screened each record and each report retrieved, whether they worked independently, and if applicable, details of automation tools used in the process.	Page 8-9
Data collection process	9	Specify the methods used to collect data from reports, including how many reviewers collected data from each report, whether they worked independently, any processes for obtaining or confirming data from study investigators, and if applicable, details of automation tools used in the process.	Page 9
Data items	10a	List and define all outcomes for which data were sought. Specify whether all results that were compatible with each outcome domain in each study were sought (e.g. for all measures, time points, analyses), and if not, the methods used to decide which results to collect.	Page 8-9
	10b	List and define all other variables for which data were sought (e.g. participant and intervention characteristics, funding sources). Describe any assumptions made about any missing or unclear information.	Page 8-9
Study risk of bias assessment	11	Specify the methods used to assess risk of bias in the included studies, including details of the tool(s) used, how many reviewers assessed each study and whether they worked independently, and if applicable, details of automation tools used in the process.	Page 9-10
Effect measures	12	Specify for each outcome the effect measure(s) (e.g. risk ratio, mean difference) used in the synthesis or presentation of results.	Page 8

Section and Topic	Item #	Checklist item	Location where item is reported
Synthesis methods	13a	Describe the processes used to decide which studies were eligible for each synthesis (e.g. tabulating the study intervention characteristics and comparing against the planned groups for each synthesis (item #5)).	Page 9
	13b	Describe any methods required to prepare the data for presentation or synthesis, such as handling of missing summary statistics, or data conversions.	Page 9
	13c	Describe any methods used to tabulate or visually display results of individual studies and syntheses.	Page 10
	13d	Describe any methods used to synthesize results and provide a rationale for the choice(s). If meta-analysis was performed, describe the model(s), method(s) to identify the presence and extent of statistical heterogeneity, and software package(s) used.	Page 10
	13e	Describe any methods used to explore possible causes of heterogeneity among study results (e.g. subgroup analysis, meta-regression).	Page 10-11
	13f	Describe any sensitivity analyses conducted to assess robustness of the synthesized results.	Page 10-11
Reporting bias assessment	14	Describe any methods used to assess risk of bias due to missing results in a synthesis (arising from reporting biases).	Page 11
Certainty assessment	15	Describe any methods used to assess certainty (or confidence) in the body of evidence for an outcome.	Page 9-10
**RESULTS**			
Study selection	16a	Describe the results of the search and selection process, from the number of records identified in the search to the number of studies included in the review, ideally using a flow diagram.	Page 11 (figure 1)
	16b	Cite studies that might appear to meet the inclusion criteria, but which were excluded, and explain why they were excluded.	Page 11 (figure 1)
Study characteristics	17	Cite each included study and present its characteristics.	Page 11 (table 1)
Risk of bias in studies	18	Present assessments of risk of bias for each included study.	Page 12 (Supplementary table S2)
Results of individual studies	19	For all outcomes, present, for each study: (a) summary statistics for each group (where appropriate) and (b) an effect estimate and its precision (e.g. confidence/credible interval), ideally using structured tables or plots.	Page 12 (Table 1)
Results of	20a	For each synthesis, briefly summarise the characteristics and risk of bias among contributing studies.	Page 12 (Supplementary

Section and Topic	Item #	Checklist item	Location where item is reported
syntheses			table S2)
	20b	Present results of all statistical syntheses conducted. If meta-analysis was done, present for each the summary estimate and its precision (e.g. confidence/credible interval) and measures of statistical heterogeneity. If comparing groups, describe the direction of the effect.	Page 12 (Figure 2a and 2b)
	20c	Present results of all investigations of possible causes of heterogeneity among study results.	Page 12-13 (Supplementary table S4 and supplementary figures S1-9)
	20d	Present results of all sensitivity analyses conducted to assess the robustness of the synthesized results.	Page 12-13 (Supplementary table S4 and supplementary figures S1-9)
Reporting biases	21	Present assessments of risk of bias due to missing results (arising from reporting biases) for each synthesis assessed.	Page 13 (Figure 4)
Certainty of evidence	22	Present assessments of certainty (or confidence) in the body of evidence for each outcome assessed.	Page 13 (Figure 4)
**DISCUSSION**			
Discussion	23a	Provide a general interpretation of the results in the context of other evidence.	Page 13-16
	23b	Discuss any limitations of the evidence included in the review.	Page 17-18
	23c	Discuss any limitations of the review processes used.	Page 17-18
	23d	Discuss implications of the results for practice, policy, and future research.	Page 18
**OTHER INFORMATION**			
Registration and protocol	24a	Provide registration information for the review, including register name and registration number, or state that the review was not registered.	Abstract<br>Page 6
	24b	Indicate where the review protocol can be accessed, or state that a protocol was not prepared.	Abstract<br>Page 6
	24c	Describe and explain any amendments to information provided at registration or in the protocol.	Page 6

Section and Topic	Item #	Checklist item	Location where item is reported
Support	25	Describe sources of financial or non-financial support for the review, and the role of the funders or sponsors in the review.	NA
Competing interests	26	Declare any competing interests of review authors.	NA
Availability of data, code and other materials	27	Report which of the following are publicly available and where they can be found: template data collection forms; data extracted from included studies; data used for all analyses; analytic code; any other materials used in the review.	NA

Supplementary table S6: PRISMA checklist

**Project title**	Association between ultra-processed food intake and development of non-alcoholic fatty liver disease: A Systematic Review and Meta-Analysis
**First reviewer**	Dr Alex Henney
**Supervisor**	Professor Dan Cuthbertson

**1. Background to review**
Non-alcoholic fatty liver disease (NAFLD) is a non-communicable disease that exists on a continuum ranging from hepatic steatosis to inflammatory non-alcoholic steatohepatitis (NASH). The journey along the continuum exists with progressive risk of developing cirrhosis and consequent end stage liver disease or hepatocellular carcinoma (HCC) (Chalasani et al., 2012). NAFLD is identified via liver biopsy and histology, with confirmatory changes to hepatocytes including increased triglyceride deposition within lipid droplets in the absence of other causative agents such as alcohol (Sunny et al., 2017). It is now considered the hepatic component of metabolic syndrome (MetS) and is known to be associated with other diseases that are contained within the MetS umbrella including type 2 diabetes mellitus (T2DM) and obesity (Mantovani et al., 2021, Lu et al., 2018). As is seen in MetS, NAFLD has a growing incidence and prevalence affecting up to a third of adults have evidence of NAFLD (Younossi et al., 2016); making it the most

common hepatic disease globally (Estes et al., 2018). Furthermore, similarly to T2DM and obesity, prevalence of NAFLD appears to be correlated with socioeconomic status (Anstee et al., 2019).

A probable contributing factor to the increasing disparities in prevalence seen between socioeconomic groups is a dramatic transformation in the global food system with rapid growth of ultra-processed food (UPF) consumption (Monteiro et al., 2013). UPFs are industrial formulations of cheap ingredients from high yield crops such as refined sugar, starch, oil, protein isolates and remnants of intense animal agriculture that are highly energy dense due to total fat, saturated fat and trans-fat contributions, alongside containing low fibre and micronutrient profiles (Monteiro et al., 2019). They include a variety of products such as cookies, confectionary sweets, high-sugar drinks and 'microwave ready-meals'. These foods are now making up around half of total daily energy intake in western populations (Baker et al., 2020). The cheap production cost of these products, in combination with the higher relative cost of minimally processed foods, is what is driving the high consumption rate globally; particularly in low income households (Newton et al., 2017). A myriad of food processing classification systems have

been developed to assess the processing level of food. A recent systematic review highlighted that NOVA is the most specific, coherent, clear, comprehensive and workable of these systems (Moubarac et al., 2014).

To date, meta-analyses have demonstrated positive associations between UPF consumption and development of overweight and obesity (Askari et al., 2020), T2DM (Delpino et al., 2022), cardiovascular disease (CVD) and overall cancer risk (Chen et al., 2020, Lane et al., 2021). In addition, experimental study has demonstrated that UPFs provide fertile ground for promoting inflammatory diseases (Zinöcker and Lindseth, 2018), observational study has demonstrated evidence to support the link between UPF and NAFLD (Zhang et al., 2022, Ivancovsky-Wajcman et al., 2021, Konieczna et al., 2022a, Fridén et al., 2022) and poor diet quality is a known predictor of NAFLD (Ma et al., 2018). However, no meta-analyses to date have objectively explored the association between UPF and NAFLD.

In light of the above, this current review aims to assess the relationship between UPF and NAFLD and quantify this through meta-analysis.

2. **Specific objectives**
- To assess the relationship between UPF and NAFLD through systematic review of existing literature<br>- To quantify the relationship between UPF and NAFLD through meta-analysis of included data<br>- To assess the dose-response effect of the relationship between UPF and NAFLD through meta-analysis

3. **Inclusion criteria – PICOS**	
**Population**	- Adult patients with NAFLD<br>- Any severity of NAFLD from hepatic steatosis to NASH<br>- Any gender, any race<br>- Not restricted to UK but paper needs to be written in English
**Intervention/exposure**	- UPF consumption<br>- Classified by NOVA system<br>- Assessed through dietary assessment tools
**Control**	Diets that are more minimally processed
**Outcome**	Development of NAFLD; from hepatic steatosis to NASH<br>Assessed using any of the following:<br>- Quantification of liver fat via magnetic resonance imaging or magnetic resonance spectroscopy

	- Steatosis score<br>- Liver biopsy measures
**Study design**	- Longitudinal design<br>- Observational studies<br>- Must use OR, HR or RR ± CI

4. **Exclusion criteria**
- Animal studies<br>- In vitro studies<br>- Secondary research including other review articles<br>- Cross-sectional analysis<br>- Paediatric population (<18 years of age)<br>- Duplicates

5. **Search methods**	
**Electronic databases**	Ovid Medline
**Other methods**	Reference checking<br>Contacting experts in field

6. **Review methodology**	
**Details of methods**	Two main reviewers<br>Disagreements resolved by discussion<br>Agree on the inclusion and exclusion criteria beforehand
**Quality assessment**	Protocol will define the method of literature critique/appraisal

	Newcastle Ottawa Scale will be used to assess the quality of evidence in the included studies<br>NutriGrade tool will be used to assess the overall credibility of evidence from the meta-analysis
**Data extraction**	Covidence will be used to store and extract data.<br><br>There will be three stages to extraction:<br>- title screening<br>- abstract screening<br>- full text screening<br><br>Reviewer number one (AH) will review first, followed by reviewer number 2 (CG). If necessary, disagreements will be resolved by discussion between. Agreed inclusion and exclusion criteria decided beforehand to help guide the extraction process; based on PICOS.<br><br>Inclusion criteria are as follows:<br>(1) were observational studies<br>(2) considered ultra-processed food as the exposure (as defined by the NOVA classification system)

	(3) examined the association with NAFLD<br>(4) reported data as odds ratio, relative risks, or hazard ratio with corresponding 95% CIs for the association of UPF consumption with excess NAFLD<br>(5) were published in English.<br>(6) Adults<br><br>Exclusion criteria are as follows:<br>(1) Animal studies<br>(2) In vitro studies<br>(3) Secondary research including other review articles<br>(4) cross-sectional studies<br>(5) Non-adult populations<br>(6) duplicates<br><br>The data to be extracted from included papers includes the first author's name, year of publication, country and setting of the study, number of participants, age, gender, follow-up duration, methods for evaluating exposure, study main findings and covariates applied

	for adjustments in the multivariable analyses.<br><br>Specifically, this is to include any of the following:<br>1) serological measures of liver function<br>2) measures of liver fat, as assessed via magnetic resonance imaging or magnetic resonance spectroscopy<br>3) steatosis score<br>4) liver biopsy measures<br>5) blood lipid levels<br>6) measures of glycemic control<br>7) measures of body mass/composition<br>8) Other cadiometabolic risk factors<br>9) UPF consumption<br><br>Data will be recorded on excel. It is to include units for the outcome measure, the number of individuals in each group on which the analysis was based, the baseline measure and its variability, the end-of-treatment measure and its variability, the change from baseline measure and its variability, and the results of statistical analyses, both within groups and between groups.

**Systematic review**	Narrative synthesis will be done alongside meta-analysis and will be carried out using a framework which consists of four elements:<br>- Developing a theory of how the exposure works, why and for whom<br>- Developing a preliminary synthesis of findings of included studies<br>- Exploring relationships within and between studies<br>- Assessing the robustness of the synthesis
**Meta-analysis**	The data will be transcribed into Excel spreadsheets. It is to include the study authors, date of publication, measure of increased risk of NAFLD following both moderate and high consumption of UPF. We will also include n= for the outcome of NAFLD, the number of individuals in each group on which the analysis was based, the baseline measure and its variability (95% CI), the end-of-treatment measure and its

	variability, the change from baseline measure and its variability, and the results of statistical analyses, both within groups and between groups.<br><br>A random effects model will be used to calculate a pooled RR ± 95% CI for all the included studies. This will be conducted using R Studio software. The Higgins I^2 statistical technique will be used to assess heterogeneity between the included studies (Higgins and Thompson, 2002). To be considered highly heterogenous, values will be required to be above 50% with a p value <0.05.<br><br>Funnel plots will be generated and Eggers test will be performed to quantify better publication bias.<br><br>For the purpose of this Meta-Analysis, all values will be converted to RR. HR values were considered equivalent to RR. OR values were converted to RR using the following equation:

	$RR = OR/[(1-P_o) + (P_o \times OR)]$, in which P_o represents the incidence of the outcome of interest in the non-exposed group (Zhang and Yu, 1998).
**Scoring evidence**	Newcastle Ottawa Scale<br>NutriGrade tool
**7. Presentation of results**	
**Additional material**	- Protocol<br>- Flow chart of whole process Data extraction form and tables<br>- Funnel plot to visually highlight publication bias<br>- Forest plots of studies included in the final review
**Outputs**	- 1 paper in high quality obesity journal<br>- Conference presentations
**8. Timeline**	
**Protocol**	Week 1
**Literature search**	Week 3
**Quality appraisal**	Week 4
**Data extraction**	Week 5

**Synthesis**	Week 7
**Writing up**	Week 9

Supplementary table S7: Original study protocol
